# Sensor-Based Human Activity Recognition with Spatio-Temporal Deep Learning

**DOI:** 10.3390/s21062141

**Published:** 2021-03-18

**Authors:** Ohoud Nafea, Wadood Abdul, Ghulam Muhammad, Mansour Alsulaiman

**Affiliations:** 1 Department of Computer Science, College of Computer Science and Engineering, Taibah University, Medina 42353, Saudi Arabia; 2 Department of Computer Engineering, College of Computer and Information Sciences, King Saud University, Riyadh 11543, Saudi Arabia; aabdulwaheed@ksu.edu.sa (W.A.); mmalsulaiman@ksu.edu.sa (M.A.); 3 Center of Smart Robotics Research, College of Computer and Information Sciences, King Saud University, Riyadh 11543, Saudi Arabia

**Keywords:** human activity recognition, local spatio-temporal features, deep learning, convolution neural networks, Bi-directional LSTM

## Abstract

Human activity recognition (HAR) remains a challenging yet crucial problem to address in computer vision. HAR is primarily intended to be used with other technologies, such as the Internet of Things, to assist in healthcare and eldercare. With the development of deep learning, automatic high-level feature extraction has become a possibility and has been used to optimize HAR performance. Furthermore, deep-learning techniques have been applied in various fields for sensor-based HAR. This study introduces a new methodology using convolution neural networks (CNN) with varying kernel dimensions along with bi-directional long short-term memory (BiLSTM) to capture features at various resolutions. The novelty of this research lies in the effective selection of the optimal video representation and in the effective extraction of spatial and temporal features from sensor data using traditional CNN and BiLSTM. Wireless sensor data mining (WISDM) and UCI datasets are used for this proposed methodology in which data are collected through diverse methods, including accelerometers, sensors, and gyroscopes. The results indicate that the proposed scheme is efficient in improving HAR. It was thus found that unlike other available methods, the proposed method improved accuracy, attaining a higher score in the WISDM dataset compared to the UCI dataset (98.53% vs. 97.05%).

## 1. Introduction

As a significant discipline of study in computer vision, human activity recognition (HAR) has applications in fields ranging from human–computer interaction to healthcare. With advancing technologies, such as camera devices and imaging techniques, novel HAR modalities are continuously emerging. Given its ability to yield high-level insights into human activity from raw sensor inputs, HAR is used in areas such as gait analysis, gesture recognition, video surveillance, and home behavior analysis. Video-based HAR, which examines videos or images that include human motion, and sensor-based HAR, which uses smart sensor data (e.g., accelerometers, sound sensors, or gyroscopes), are the two main categories of HAR. With the increasing ubiquity of smart sensor technology and the availability of robust cryptosystems for ensuring data privacy, sensor-based HAR is growing in popularity.

Various types of sensors have been investigated for their potential to make activity detection more precise. Thus, fixed sensors and mobile sensors have emerged as the basis for two different types of methods for detecting human activity, depending on the way in which sensors are used within a setting. Mobile sensor-based methods make use of specialized movement sensors placed on the body (e.g., accelerometers, gyroscopes, magnetometers) to collect data on various activities. Human activities can be extracted from data on acceleration and angular velocity because they alter in keeping with human movements. Furthermore, unlike fixed sensors, mobile sensors are small in size and flexible, so that they can be incorporated in body gear or mobile devices. Mobile sensors are additionally advantageous as they are inexpensive, use less energy, have high capability, and are more independent of the environmental setting. Hence, the extensive incorporation of mobile sensors in everyday life is the reason for the increased interest in mobile sensor-based activity detection, with numerous studies having been dedicated to the investigation of the suitability of mobile sensors for identifying human activities [1].

**Figure 1:**
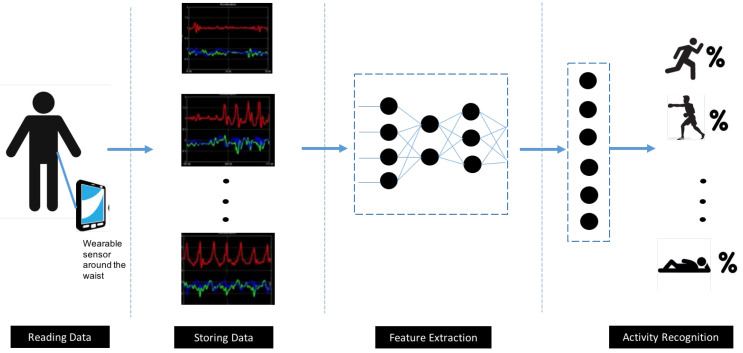
A block diagram of sensor-based human activity recognition using deep learning.

Detection of human activities based on mobile device sensors has been typically recognized as a problem of multivariate time series classification. Feature extraction is a major stage in addressing the problem and can be achieved based on certain raw signal statistical aspects, such as variance, mean, entropy, and correlation coefficients, or through integration of cross-formal coding, such as signals with Fourier transform and wavelet transform. Detection of various types of activities has been successfully achieved via conventional machine learning methods, including decision tree, support vector machine, and naïve Bayes [2]. On the other side, hand-crafted feature extraction often underpins such methods and demands expertise or knowledge of domains. Automatic feature extraction is possible within a deep-learning setting via the designing of a deep model with multiple layers [3]. These approaches can only learn shallow features, which reduces performance levels for incremental and unsupervised tasks. In view of these challenges, conventional pattern recognition methods tend not to have high classification accuracy or model generalization [4]. Human activity recognition consists of several stages. It starts with time series data pre-processing and segmentation, followed by data feature extraction, and application of a relevant algorithm for classification purposes as illustrated in Figure 1.

The most significant development in machine learning recently has been convolution neural networks (CNN). These models can be used instead of methods with manual feature extraction and have led to an increase in pre-eminence of network engineering over feature engineering. Furthermore, there is a growing emphasis on the development of smaller network configurations without altering performance. There is evidence that the designing of CNNs of greater depth can enhance performance for various HAR tasks, although at the expense of a larger number of resources (e.g., memory, computational power) [2]. Moreover, resource restrictions make it impractical to integrate deep models in mobile devices because their parameters typically number in their millions, so deep learning for HAR cannot be applied widely in the case of mobile devices [2]. This calls for the creation of lightweight CNNs.

Growing attention has been paid to the development of CNNs of small size and high efficiency for different embedded applications intended to minimize the number of parameters. Regarding this, research has been spearheaded by computer vision studies, sparking further investigations on lightweight network design for using of pre-trained networks or designing of small networks from scratch [2]. However, the adaptation of this approach to HAR tasks remains to be studied. The use of sensors by HAR can basically be construed as a standard problem of multivariate time series classification, whereby sensor signals are segmented via a sliding window and differentiating features are extracted from those signals for the purpose of activity recognition via a classifier. Hence, the difficulties confronting the HAR task sets it apart from imagery data. Outstanding outcomes have been accomplished in computer vision tasks based on lightweight network modules, yet such modules have not been often used in relation to HAR. Several studies have explored the feasibility of re-designing filters and its kernel in deep learning, since the filter is the fundamental component in CNN development. Thus, the use of a set number of filters with different kernel size can capture different aspects of data in sensor-based HAR.

Nevertheless, no single deep-learning model can overcome all the issues related to HAR. For instance, suitable local features can be extracted from sensor data highly efficiently by CNNs, yet these models lack memory and overlook temporal dependencies among data records. By contrast, problems in which temporal dependencies are significant can be successfully addressed with recurrent neural networks (RNNs). Long short-term memory (LSTM) exhibit significantly higher performance compared to conventional RNNs in terms of long-term memory of dependencies owing to the structure of its repeating module [5]. Overall, deep-learning models are most useful because they can learn complicated features from unprocessed data, so that a priority knowledge and hand-crafted extraction of features are not required.

CNN with varying kernel dimension along with bi-directional long short-term memory (BiLSTM) has been proposed as a new deep neural network for human activity detection to overcome problems presented by the above-mentioned approaches. The proposed model was capable of automatic extraction of activity attributes and subsequent categorization of those attributes using a handful of parameters. Therefore, the approach put forth in this research employs a technique that does not require statistical features to be applied to raw signal data. Furthermore, both spatial and temporal features are considered in feature extraction. Two common public datasets were employed for assessing the model, which was found to be highly accurate, while also demonstrating satisfactory generalization and rapid speed of convergence. The remainder of this paper provides an overview of existing related works and explains in detail the proposed model. The reminder of this paper is organized as follows. The most common modalities of sensors and a brief background about previous studies in the field of HAR is given in Section 2. A detailed description of the proposed approach is described in Section 3. The experimental setting and the obtained results are discussed in Section 4. In Section 5, we conclude our proposed work and discus the main limitations of the proposed approach.

## 2. Background

Although it is possible to generalize certain HAR approaches to every sensor modality, most are specific and narrow. There are three types of modalities: body-worn sensors, ambient sensors, and object sensors.

### 2.1. Types of Sensor Modalities

#### 2.1.1. Body-Worn Sensors

Sensors worn on the body (i.e., body-worn sensors such as gyroscopes, magnetometers, and accelerometers) are a prevalent HAR modality. These devices can gather data on human activities based on changes in angular velocity and acceleration. Body-worn sensors have been used in deep learning for HAR in several studies, most of which have focused on data obtained from accelerometers. To recognize activities of daily living (ADL) as well as specific activities related to sports, gyroscopes and magnetometers are often combined with accelerometers [6].

#### 2.1.2. Ambient Sensors

Ambient sensors, which are typically integrated into a user’s smart environment and include radar, temperature sensors, pressure sensors, and sound sensors, are often used to gather data on interactions between humans and the environment. Various object sensors are used to measure object movements, and ambient sensors capture environmental changes. Several studies have used ambient sensors for HAR in ADL and hand gestures [6].

#### 2.1.3. Hybrid Sensors

Several studies have used acceleration or sensors with ambient sensors to optimize HAR accuracy. It indicates that the use of hybrid sensors, establishing varied datasets from multiple sources, can significantly aid research efforts in HAR, and promote applications in applications such as commercial smart home systems [6].

### 2.2. Deep-Learning Models

The purpose of this section is to provide an overview of some deep-learning models that are applied in HAR.

#### 2.2.1. Convolution Neural Networks (CNN)

CNNs rely on sparse interactions, equivariant representations, and parameter sharing. Following convolution, fully connected (FC) and pooling layers are generally employed for regression or classification. Compared with other models in terms of performance when used for time series classification in HAR, CNNs benefit from both scale invariance and local dependency. Owing to the efficacy of CNNs, most research has focused on this topic. For CNN application to HAR, a range of considerations must be considered, including pooling, weight-sharing, and input adaptation [4].

#### 2.2.2. Autoencoder

Latent representations of input values are learned by autoencoders via hidden layers, which can be regarded as an encoding-decoding process. An encoder learns advanced features based on an unsupervised learning approach. Stacked autoencoders (SAEs) treat each layer as the autoencoder’s basic model, and following multiple training periods, learned features are stacked with labels to create a classifier. A critical benefit associated with SAE is the ability to undertake unsupervised feature learning in HAR, which could prove valuable in feature extraction. However, the high level of dependency on layers and activation functions in SAE may create situations in which it is difficult to identify an ideal solution.

#### 2.2.3. Restricted Boltzmann Machine (RBMs)

Restricted boltzmann machine (RBMs) are fully connected (FC), bipartite, undirected graphs that include hidden and visible layers [7]. Stacked RBMs are referred to as deep belief networks (DBNs), which can be established by treating every two consecutive layers as a single RMB. DBN/RBM is frequently followed by FC layers [8].

#### 2.2.4. Recurrent Neural Network (RNN)

RNNs are frequently employed for natural language processing and speech recognition. Successes have been achieved in these areas due to the exploitation of temporal connections existing between neurons (or nodes) in a network. RNNs are commonly used with LSTM cells to enable memory through gradient descent. Relatively few prior studies have used RNNs for HAR, but in these previous studies, the speed of learning and computational overhead were the key issues in HAR. As the literature indicates, the key issue in RNN-based HAR models is to satisfy requirements in low computing power settings without undermining performance [9].

#### 2.2.5. Hybrid Model

Hybrid models refer to models that combine several deep-learning models. One example involves combining CNNs and RNNs. This combination can be performed well because CNNs and RNNs reflect spatial and temporal relationships, respectively. Hence, in terms of recognizing activities with diverse signal distributions and time spans, the use of CNNs and RNNs together could promote increases in performance. Other studies have used CNNs with stacked autoencoders (SAE) and RBM. In these studies, CNN performed feature extraction, while the generative models increased the speed of the training phase [4].

A range of challenges is associated with HAR, including the issue of the dynamic, complex background, the change in perspective, the camera’s height, and variability in the morphological features of human beings. Additionally, significant variability both within and between activity categories represents a critical challenge for continued progress in the field [10]. Figure 1 presents a block diagram of sensor-based HAR. In the next section, a summary of prior studies addressing the HAR pipeline is given.

With the development of deep-learning techniques, significant advances have been achieved in fields such as natural language processing, visual object recognition, and logical reasoning. The key difference between conventional pattern recognition methods and deep learning is that the latter can avoid having to expend significant effort on designing features, and it can learn more meaningful, high-level features through training on an end-to-end (E2E) neural network. With these considerations in mind, deep-learning is appropriate for HAR, and as such, many scholars have already examined the possibilities in this area [11].

HAR is significantly shaped by temporal movement, which is overlooked by the deep-learning CNN model. The principal neural network approaches suitable for HAR that yield promising outcomes in terms of HAR based on sensor information from fitness devices and smartphones are recurrent neural network (RNN) models and CNN models [12]. This combination between CNN and RNN can extract the temporal and spatial features effectively. In this study, we designed a temporal and spatial deep-learning model as a form of two-stream deep-learning infrastructure that allows deep-learning techniques to obtain spatial and temporal features using CNN and BiLSTM models.

### 2.3. Literature Review

There are two approaches for HAR: first, manual feature extraction; and second, automatic feature extraction, which relies on deep-learning. Here, we explore HAR, focusing on deep-learning approaches.

The convolutional architectures in video-based action recognition have been proposed in several studies such as in [13,14]. Using a two-stream ConvNets, namely VGGNet and GoogLeNet is conducted by [13]. Obtaining dynamic images through the direct application of rank pooling on a video’s raw image pixels, which generates one RGB image for every video is conducted by [14]. The concept is straightforward, but significant, mainly because it allows available CNN models to be directly applied to video data with some particular configuration. Ronao et al. used CNN to recognize the activities that were recorded by sensors [15]. Feichtenhofer et al. [16] formulated another spatio-temporal architecture for a pair of stream networks, each of which had a new convolutional fusion layer between the networks. The well-known issue of the proposed two-stream ConvNets relates to their weaknesses in modelling long-range temporal structure, as discussed in the study conducted by [17]. This stems from their lack of access to temporal context, as they were only ever intended to function on one frame (spatial networks) or one stack of frames in a brief snippet (temporal network).

One of the developed versions of CNN is designing a lightweight CNN for HAR based on Lego filters that was proposed by Tang et al. [2]. Author’s in [18] have proposed a multi-stage transformation of a raw signal data. Diverse representations of the raw data-encoded features are obtained at first by subjecting time series data to a different number of transformations, rather than just one transformation. Individual training for extraction of features from various transformed spaces is applied with an effective deep CNN architecture. A basic issue in these approaches that it required a prior knowledge of features.

Author’s in [19] designed a trajectory-pooled deep-convolutional descriptor (T-DD) to leverage the benefits of both deep-learning-derived and hand-crafted features. Another work by Ignatov [20] used CNN along with statistical features to preserve both local and global features. Encoding the features as images from ten types of indoor activity class were obtained and pre-processed to match the data format, after which they were converted into 2D feature vectors with the log Mel-band energy technique [21]. Most of the studies that employ only a 2D-CNN to extract the features ignore the concept of translation in time between frames which is a significant factor to identify the action. Also, the image quality, illumination, brightness and camera setting are affected the performance of these systems.

The aim of Tran et al. [22] was to solve the issue of learning spatio-temporal features of videos with 3D-ConvNets. Carreira and Zisserman [23] proposed a two-steam inflated 3D-ConvNet (I3D). This method relied on 2D ConvNet inflation, particularly in terms of the use of filters and pooling kernels for deep image classification. 3D-ConvNets increased in size by adding another dimension, meaning that seamless spatio-temporal feature extractors could be captured from the video, at the same time exploiting effective ImageNet architectures and parameters. The study’s results indicated that following pre-training on kinetics, I3D models showed substantial improvements in terms of action classification.

Wang et al. [24] proposed that a CNN could be used to extract only the spatial features for every frame, and so the researchers designed two types of LSTM networks to examine temporal features in successive video frames. In Xia et al. [1], mobile sensors were used to acquire raw data, which were introduced in an LSTM two layers, with subsequent convolutional layers. Furthermore, the model parameters were restricted by substituting the fully linked layer with a global average-pooling layer. CNNs and LSTMs in activity recognition in younger adults has been widely studied, their function in older people had been largely under-investigated. Thus, in the research presented by Nan et al., they improved many models such as 1D CNN, a multichannel CNN, a CNN-LSTM, and multichannel CNN-LSTM model. Comparisons were made between the computational efficiency and accuracy of the different models and ultimately, the well-developed multichannel CNN-LSTM model was found to be the most suitable approach for investigation long-term activity recognition in older individuals [25].

In the study conducted by Sargano et al. [24], the researchers found that to apply techniques that rely on deep learning (e.g., those involving 3D-CNN or LSTM networks), a large data set is required to perform the learning. Hence, certain models are unsuitable for small data sets due to the challenge arising from overfitting in CNNs. Methods reliant on learning spatial features with pre-trained deep neural networks, they do not address temporal features, but have better performance than current reference methods because these methods leverage the pre-trained model’s knowledge. A configuration integrating a shallow CNN for unsupervised extraction of local features and statistical features encoding universal attributes related to the time series was put forth in a study by Ignatov [20]. The impact of the length of time series on detection precision was examined as well. A summary of these proposed approaches is listed in Table 1. Based on these findings, a HAR technique is proposed in this research that relies on an E2E deep neural network.

## 3. Proposed Approach

**Figure 2:**
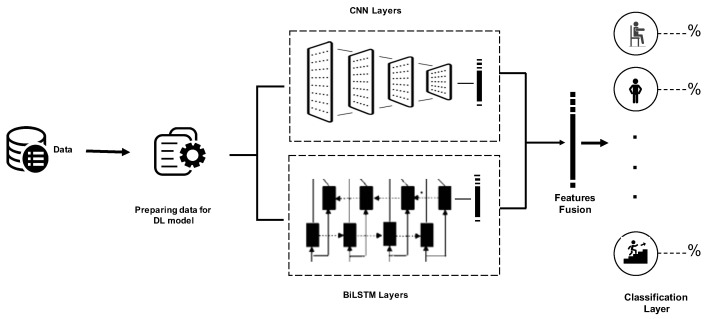
Overall architecture of the proposed approach.

In the proposed approach, we will work to develop an E2E temporal and spatial deep-learning model as a form of two-stream deep-learning architecture based on CNN and BiLSTM that allows deep-learning techniques to obtain temporal cues. Subsequently, activity recognition is completed through the fusion of features that was obtained from both streams. Figure 2 presents the overall architecture, with the spatial and temporal characteristic streams depicted, respectively. The inherent temporal relationship of the spatial deep-learning map is understood via BiLSTM. A new architecture of the spatial stream with many convolution layers and with varying kernel dimensions was used to achieve feature capture at various resolutions. Below, we explain the basic structure of CNN and BiLSTM as well as the basic metrics used to evaluate deep-learning models.

### 3.1. Basic Structure of CNNs

A specific set of components are generally applied when using the CNN. Supervised learning often uses CNNs. These neural networks usually connect each neuron to every other neuron in each subsequent layer of the network itself. The neurons’ input value is transformed into the output value using the activation function of the neural network. The activation function has two notable factors that govern its quality. These are sparsity and its ability to handle the reduced gradient flow to the neural network’s lower layers [26]. CNNs often use pooling as a form of dimensionality reduction. Both the maximum and average-pooling functions are typically used, which are referred to as max- and average-pooling, respectively.

### 3.2. Basic Structure of Long Short-Term Memory (LSTM)

LSTM architecture has proven to be effective to obtain temporal information concerning HAR [21]. LSTM unit can determine whether existing memory should be retained, or new information should be added to it. Hence, LSTM-RNN can create long-range dynamic dependencies for avoiding the vanishing or exploding gradients problem while training. Regarding the time series classification, the principal elements of an LSTM network include the sequence input layer, the LSTM layer, the FC layer, the SoftMax layer, and the classification output layer. More details can be found in Rashid and Louis [27].

#### 3.2.1. Training LSTM

**Figure 3:**
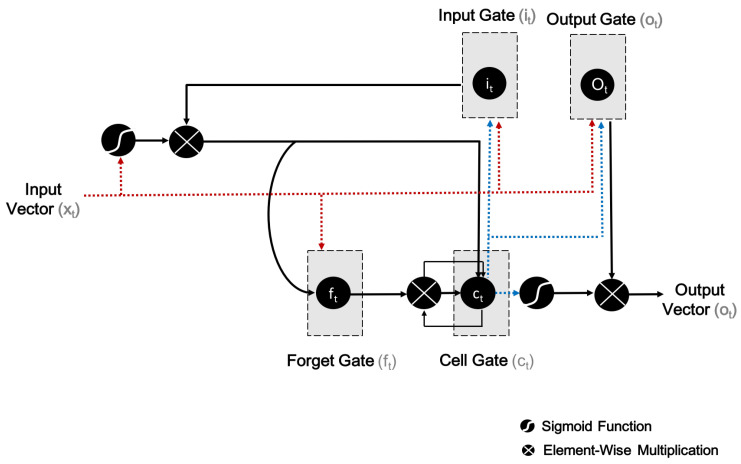
Architecture of a long short-term memory (LSTM) unit.

The LSTM layer comprises of LSTM units, as well as a shared architecture of such units, namely an input gate (*i*), output gate (*o*), cell (*c*), and forget gate (*f*). The architecture of an LSTM unit is shown in Figure 3.

Drawing on an activation function of the weighted sum, the computation of an activation function is the responsibility of each output. This refers to the activation of the *i* gate at the *t*^th^ time step, while $f_{t}$ and $o_{t}$ are the same for the *f* and *o* gates. Additionally, $h_{t}$ and $x_{t}$ denote the LSTM unit’s output and input vectors. In the case of exit arrows from c (i.e., the memory cell) to the gates, note that *i*, *o* and *f* refers to the contribution of the activation of *c* at the (t−1)^th^ step (that is to say, $c_{t-1}$).

Another way to state this is that the *f*, *i*, and *o* gates compute their activations at every *t*^th^ step, based on the *c* at the (t−1)^th^ step. In Figure 3, element-wise multiplication with respect to inputs is indicated by the circle marked with an 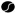, whereas the circle marked with an 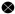 denotes the use of the sigmoid function (or, for that matter, any other application function) to a weighted sum. The LSTM network involves the automatic learning of high-level features which are related to long-term ways across time steps [27].

#### 3.2.2. Basic Structure of Bi-Directional Long Short-Term Memory (BiLSTM)

**Figure 4:**
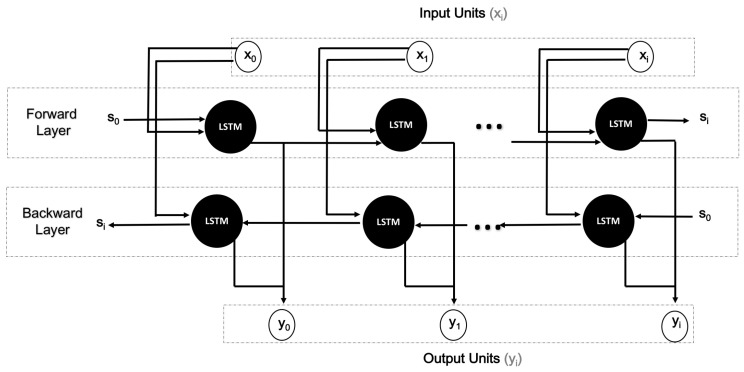
Architecture of a Bi-directional long short-term memory (BiLSTM) unit.

Figure 4 illustrates the basic structure of BiLSTM. The set of $x_{0},x_{1},x_{2},\ldots ,x_{i}$ refers to the input units, whereas $y_{0},y_{1},y_{2},\ldots ,y_{i}$ refers to the output units. The hidden units are labelled as $s_{0},s_{1},s_{2},\ldots ,s_{i}$. BiLSTM was used for obtaining the temporal representation concerning activity recognition that can access context in the forward and backward directions [28].

#### 3.2.3. Designing a Deep CNN-BiLSTM Learning Model

Employing CNN and BiLSTM to extract features is the key principle underpinning the proposed model. Here BiLSTM and CNN work in parallel as a two-stream. Subsequently, the outputs of both streams are combined to generate a final output that is used for recognition.

The structure of the system applying the proposed model is shown in Figure 2. The pre-processing module incorporated in the system underpins input data preparation for the deep-learning libraries. The CNN model subsequently undertakes processing of the segmented data. This model comprises several one-dimensional convolution layers equipped with the ReLU activation function. Feature map extraction from the input windows is undertaken by every convolution layer based on 128-size filters, each of which has a different size kernel. The CNN model additionally encompasses a max-pooling layer with 5-size pool to generate an overview of the feature maps supplied by the convolution layers and make computation more cost-effective. Furthermore, to facilitate their processing by the RNN models, the dimensions of the feature maps must be contracted as well. This involves conversion of the matrix representation of every feature map by the flatten layer into a vector. Addition of a few dropouts above the pooling layer is done for the purposes of regularization and minimization of the likelihood of overfitting. A set of neurons is overlooked by the system during training due to the use of dropouts and those neurons are chosen arbitrarily, with 0.05 probability.

**Figure 5:**
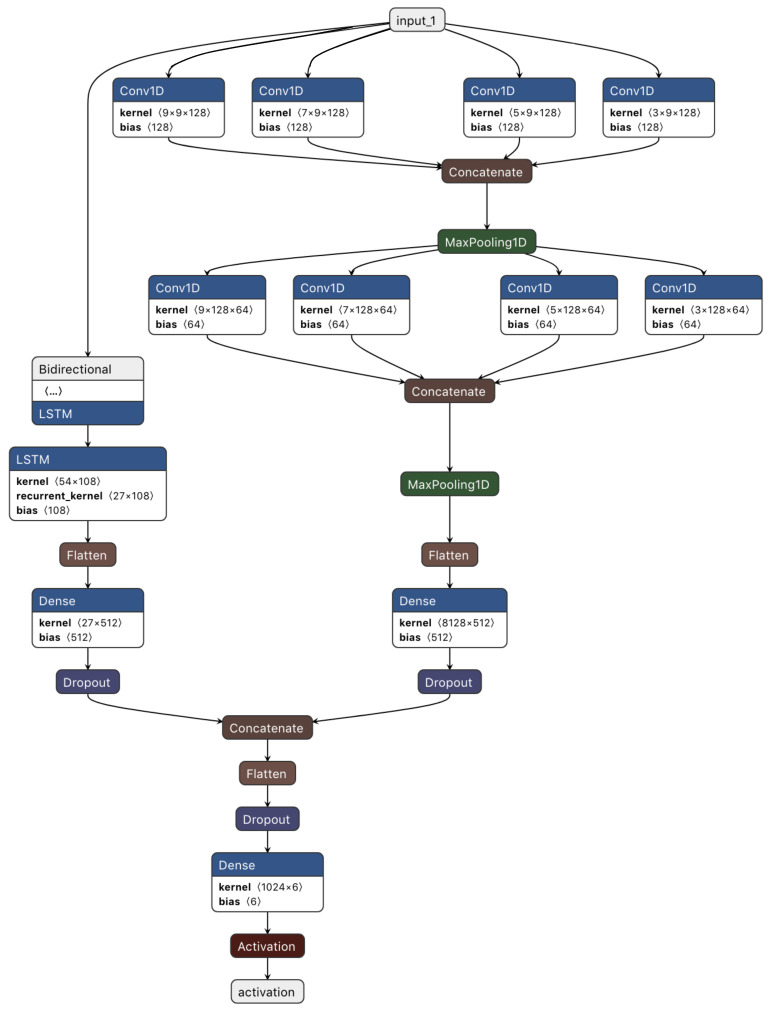
A detailed description of the proposed approach.

Figure 5 presents the detailed architecture of the proposed CNN-BiLSTM. Each convolution layer consists of a set of filters possessing the same kernel size. The kernel size is a significant parameter. This is because, to convert the input data into meaningful information, each filter’s output must respond to areas that have the same sizes as those of the different objects/patterns inputted [29]. If a filter’s kernel size is significantly larger than the input pattern, this may cause the filter to capture blurred features from the input patterns. On the other hand, if a unit’s kernel is smaller than that of the input patterns, this compromises the global structure of the input patterns [29].

Thus, to obtain meaningful information from the CNN, filters with different kernel sizes should be used in each layer. Moreover, the kernel size should be proportional to the patterns. After considering these issues, we developed the multi-resolution-CNN presented in Figure 5 with multi-resolution filters (filters with different kernel size) in each layer.

All filters in one layer are the same size. However, this size is different from those in other layers within the same set. Each set consists of four convolution layers. In Figure 5, the layers involved in the multi-resolution-CNN are presented in detail. For example, the first set has four convolution layers with four different kernel sizes. The first layer has 128 filters with a kernel size equal to 9, whereas the second layer has 128 filters with a kernel size equal to 7. The third and fourth have kernel sizes of 5 and 3, respectively. All these layers have the same size of input features, which is equal to 128×9 features, as mentioned in the following section. Multi-resolution features are created from the input features in each layer, which facilitates the production of accurate patterns that form the structure of the underlying data.

The multi-resolution-CNN involves many different parameters (including filter size, number of layers, and number of filters per set), as is the case with most deep-learning models. The selection of these parameters is usually determined by data and its application. It is a challenging task to select proper CNN parameters. In this work, the strategy used in Grais et al. [30] was followed. However, the number of filters remained constant. Additionally, as we moved through the layers, we reduced the filter sizes.

The outputs of the CNNs and the BiLSTM will then be combined. Here, the relevant temporal and spatial features can be successfully identified using the model. The conversion of the class weights determined by the earlier layers into probabilities is done by the last system layer, namely the classification layer. This layer comprises two fully connected layers, these layers subjecting its inputs to the SoftMax activation function. Weight updating and loss computation are respectively undertaken by all the system via the Adam optimization algorithm. The Adam optimizer represents an effective extension of stochastic gradient decent intended exclusively for deep artificial neural networks.

## 4. Experimental Results and Discussion

### 4.1. Database Setting

In this model, we investigated the use of sensor-based HAR using datasets such as wireless sensor data mining (WISDM) [31] and UCI-HAR dataset [32].

#### 4.1.1. Wireless Sensor Data Mining (WISDM)

Wireless sensor data mining (WISDM) dataset includes 1098209 samples. Table 2 presents the proportion of the overall samples related to every activity, revealing the unbalanced nature of the WISDM dataset. The activities of greatest and lowest prevalence are walking (38.6%) and standing (4.4%), respectively. Furthermore, 36 subjects who were required to undertake specific everyday tasks while carrying an Android phone in the front pocket of their pants constituted the experimental target of WISDM. An accelerometer with a 20-Hz sampling frequency served as the sensor, while being a motion sensor integrated in smartphones as well. Standing (Std), sitting (Sit), walking (Walk), upstairs (Up), downstairs (Down), and jogging (Jog) were the documented activities. To ensure that data were of high quality, a designated individual monitored the process of data collection. The features of the raw data on every axis could be observed based on the acceleration waveform of 2.56 s (128 overall number of points). We assigned 70% for training and 30% for testing.

#### 4.1.2. UCI-HAR

The dataset of UCI is obtained from the recordings of 30 individuals doing different activities of ADL while wearing waist-mounted smartphones with embedded inertial sensors. This database introduced a new type of data parameter and reduced the time required for training and testing the model effectively. It involved 30 participants between 19 and 48 years of age. Table 3 presents the proportion of the overall activities in UCI dataset.

All participants had a smartphone (Samsung Galaxy S II) strapped to their waist, and they all executed six activities: Sit, Walk, Walk Up, Walk Down, Std, and Laying Down. The phone’s embedded gyroscope and accelerometer were used to capture 3-axial linear acceleration as well as 3-axial angular velocity. Gyroscopic data (angular velocity) and x, y, and z accelerometer data (linear acceleration) from a Samsung Galaxy S II were used as movement data. Fifty data points were captured each second (i.e., recording of observations at 50 Hz), and every participant performed the activity sequence a total of two times: first, with the device on their left-hand side and then with the device on their right-hand side.

Several often-used frequency and time features in HAR were captured from each window. The outcome was a feature vector containing 561 elements. The primary signal types included in the raw data were the following: body acceleration, total acceleration, and body gyroscope. In each case, the signal types were associated with 3 axes of data, meaning that there were 9 variables for every time step. Every series of data were partitioned into intersecting windows (2.65 s, or 128 time steps each), meaning that a single row of data contained 1152 (128 × 9) elements. The data were then loaded into a 3D array, where the dimensions corresponded to samples, time steps, and features. To clarify, 128 time steps and 9 features were involved, and the number of samples amounted to the number of rows in each raw signal data file. The model’s output was a 6-item vector with the probability of any given window being associated with all activity types, of which there were 6. We assigned 70% for training and 30% for testing, i.e., 21 subjects for training and 9 for testing.

### 4.2. Evaluation Metrics

The evaluation procedure was carried out by dividing the dataset into two sets: training and testing. Then, the model was fitted to the training set. The prediction was made on the test set. Evaluation of the prediction was performed using metrics such as the classification accuracy, which measured the ability to recognize the activity correctly. This was defined as the number of correctly recognized activities divided by the number of activities.

Hence, performance is not correctly reflected only by the general categorization accuracy. False positives as well as false negatives are considered by the F-measure (F1 score), which integrates two measures characterized according to the overall count of samples identified accurately. Within the information retrieval community, this is referred to as “precision” and “recall”, which are respectively described as TP/TP+FP and TP/TP+FN, with TP, FP, and FN respectively denoting the number of true positives, false positives, and false negatives. As such, performance is better reflected by the F1 score compared to accuracy. Furthermore, the F1 score weighs classes according to their sample proportion, thus addressing class imbalances. The F1 score is expressed as:

(1) $$F_{1}=\sum_{i}2*w_{i}\frac{precision_{i}\cdot recall}{precision_{i}+recall}$$

Another metric used to evaluate the model is the confusion matrix. This can graphically illustrate the performance of the model. It is defined as the matrix of recognized activities versus known activities [33].

We also used recall, which finds the proportion of activities that are correctly classified, and precision, which finds the proportion of a correctly predicted activity.

### 4.3. Experimental Setup

The model training and testing were performed on a machine that has an E5-2686 v4 Xeon CPU, 32 GB RAM, and an NVIDIA V100 graphics card. The machine used an Ubuntu operating system with 32 bits.

The proposed model was developed with Python (3.7.0). We also used Theano, TensorFlow, and Keras libraries.

This model was regarded as a Sequential Keras model. One version of stochastic gradient descent, namely the efficient Adam version, was used to achieve network optimization, and since a multiclass classification problem is the central issue, categorical cross-entropy loss function was employed. The model was fitted based on a predetermined number of epochs (namely 30), and the batch size comprised 128 samples. After the fitting of the model, the evaluation occurred using a test data set, which offers insights into the accuracy of the model.

### 4.4. Discussion

**Figure 6:**
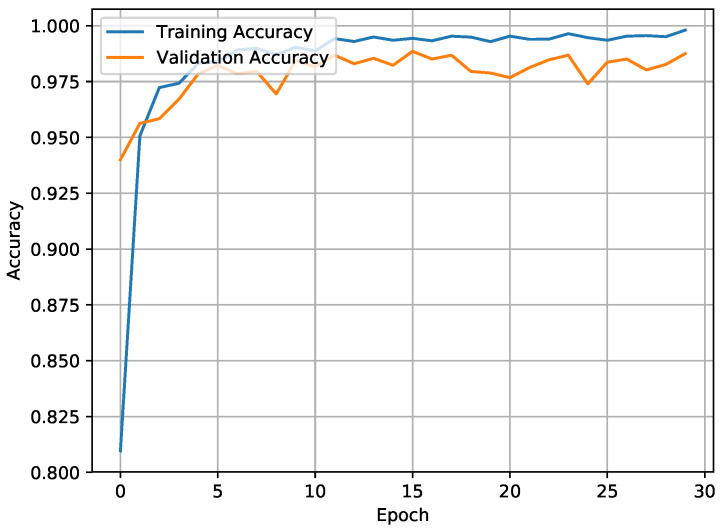
Training accuracy vs. validation accuracy.

**Figure 7:**
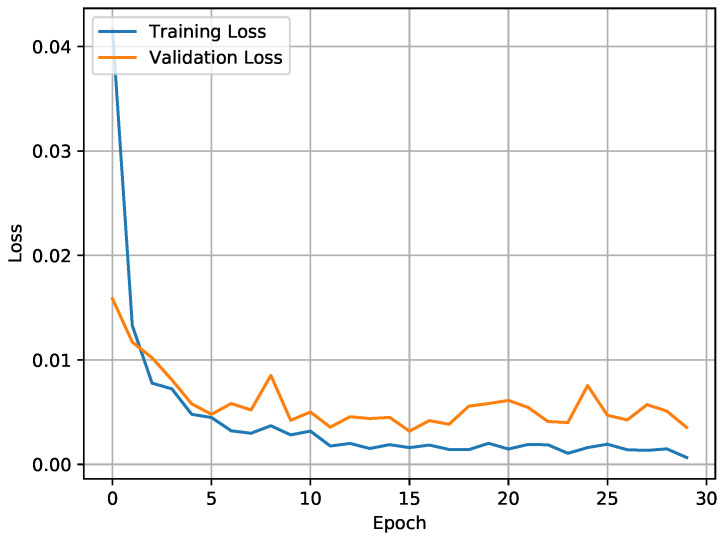
Training loss vs. validation loss.

The accuracy of the CNN-BiLSTM model was 98.53% in WISDM and 97.05% in UCI-HAR. This was an acceptable accuracy in terms of spatial and temporal features. Better training and validation accuracy was attained, with a reduction in training loss (0.0011) and validation loss (0.0086) as illustrated in Figure 6 and Figure 7.

**Figure 8:**
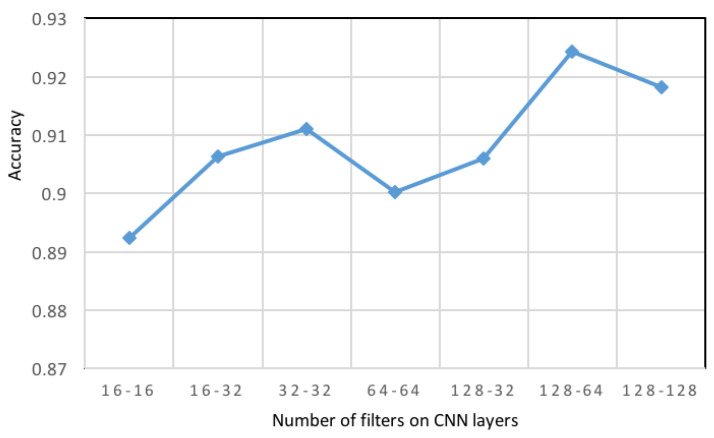
The impact of the number of filters on recognition accuracy in the proposed convolution neural networks (CNN).

Convolution kernels are capable of learning features with greater complexity. However, this may also cause overfitting due to the rise in the number of model parameters. Hence, it is essential to select the number of filters carefully. The accuracy associated with the CNN model with a different number of filters of the first and second convolution levels are illustrated in Figure 8. For 128 filters in the first level of CNN layer and 64 in the second level of CNN layer, the accuracy score was 92.43%.

The use of the UCI-HAR and WISDM test sets to estimate the model yielded the classification confusion matrices presented in Table 4, Table 5, Table 6 and Table 7. The WISDM dataset of an unbalanced nature attained a general precision of 98.53% upon exposure of the trained model to the test set. The activities of sitting and standing were not differentiated highly effectively as we have observed in Table 4, possibly due to their similarity from the viewpoint of the motion sensors. Another experiment was conducted using the proposed model, CNN-BiLSTM, as a feature extractor and support vector machine (SVM) as a classifier. We considered the extracted features at the level of concatenation layer as we illustrated the model in Figure 2. Then these features will be fed up to the SVM. The classification confusion matrix is presented in Table 5. Accurate classification using the proposed model for extraction the features and classification was achieved in 2940 instances in the case of the UCI-HAR dataset, with general accuracy of 97.05% as we observed in Table 6. Table 7 presented the confusion matrix of applying the proposed model, CNN-BiLSTM, as a feature extractor and SVM as a classifier.

Table 8 lists the performance accuracy of some studies using different datasets. In relation to the WISDM dataset, the CNN-BiLSTM performed better than other models, while in relation to the UCI dataset, the proposed method attained accuracy of 97.05%. By comparison, the maximum accuracy achieved for the UCI dataset was 97.63% via using CNN [20], Statistical features and data centering. Therefore, given that no statistical features or other hand-crafted features were employed in this work, it is not fair to compare the two approaches.

In this work, we used both types of features, spatial and temporal, and achieved an acceptable accuracy. This accuracy was obtained using the simple and effective structure of CNN and BiLSTM. However, after many experiments, we observed that the extraction of spatial and temporal features using CNN-BiLSTM was an efficient solution. In this proposed model, we maintained the relationship between the movement and spatial features. To identify a specific method concerning the intended application, different factors must be considered, and the approach should be determined accordingly; thus, although there are various methods, certain challenges continue to persist, and still require further attention.

### 4.5. Ablation Study

To validate the efficiency of the proposed work, we have conducted many experiments. For example, we have described a set of these experiments in Table 9.

## 5. Conclusions

Recently, it has become possible for deep CNNs to perform exceptionally well on different HAR benchmark datasets that necessitate a massive number of resources. However, such models are incompatible with wearable HAR sensors. Nevertheless, CNN has been computationally simplified on visual tasks using several lightweight structure designs. This research proposed a new architecture involving the use of many convolution layers, with varying kernel dimensions to achieve feature capture at various resolutions. Subsequently, temporal features were captured by applying BiLSTM in parallel.

The proposed model displays better performance compared with the earlier research on WISDM and UCI in the context of UCI use with no hand-crafted features. A comparative analysis of the confusion matrices makes it obvious that activity differentiation is most effectively undertaken by CNN-BiLSTM.

In terms of the activities of lying in UCI, 100% precision was attained by the model because those activities do not involve any motion whereas achieving 99% to recognized activities of jogging and walking in WISDM. Furthermore, sitting-up and standing-down was partially differentiated from other activities by achieving 93% and 95% respectively in UCI. Meanwhile, walking was detected well solely by the proposed model, with the others potentially confusing this activity for other similar ones, such as walking-up or waling-down. Moreover, activities with close similarities, were categorized with a high degree of precision by the proposed model.

Empirical investigation of a fusion feature at various CNN and BiLSTM levels will be considered in future studies. Another aspect warranting additional examination includes the features derived by CNN and BiLSTM through automatic extraction and their contrasting against common hand-crafted features. In relation to HAR, the main approach is deep CNN, but the attributes of the approach should be researched in greater detail and a more expansive database should be employed.

Human activity recognition can be applied to other applications such as vehicle activity recognition [34]. Autonomous vehicles represent vehicles that can guide themselves and do not rely on human drivers to manage obstacles. They are anticipated to have a higher safety performance compared to vehicles with human drivers.

## Figures and Tables

**Table 1:** Summary of the proposed studies in the related work.

Ref.	Year	Model	Domain	Proposed Study	Comments
[13]	2015	CNN	video-based	Two-stream convNets (VGGNet, GoogLeNet); 10-frame stacking of optical flow for temporal network and a single frame for spatial network; data augmentation to increase the size of the dataset	* It requires features pre-processing. * It needs the augmentation to handle the issue of overfitting. * It introduces a new representation of features and nets but it is not effectively representing the spatio-temporal features in HAR.
[14]	2016		video-based	Propose the idea of dynamic map and rank pooling to encode the video frames into a single RGB image per video.	
[15]	2016		sensor-based	Propose a multi-layer CNN with alternating convolution and pooling layers to extract the features.	
[16]	2016		video-based	Propose a two-stream network, fusion is done at the level of convolution layer.	
[17]	2016		video-based	Propose a temporal segment network (TSN); Instead of working on each frame individually it works on a set of a short snippets sparsely sampled from the video; these snippets will be fed up to a two-stream of CNN.	
[2]	2020		sensor-based	Propose to use Lego filters to have a lightweight deep CNNs	
[18]	2020		video-based	Propose to use CNN as a feature extractor from different transformed domains	
[19]	2015	CNNs with hand-crafted features	video-based	A new video representation, called trajectory-pooled deep-convolutional descriptor (TDD); two-stream ConvNets; using improved dense trajectories (iDTs) features	* It requires features pre-processing.
[20]	2018		sensor-based	Propose a new design of CNN and combine it with stats. features	* A new designing of nets but it is not effectively representing the spatio-temporal features in HAR.
[22]	2015	3D-CNN	video-based	Propose a C3D (Convolutional-3D), with a simple linear classifier	* It can capture the spatio-temporal feature more effectively than 3D-CNN.
[23]	2018		video-based	Two-steam inflated 3D-ConvNet (I3D)	
[24]	2018	CNN-LSTM	video-based	CNN to extract the spatial features, then these feature will be the input to two different stream of LSTM(FC-LSTM, ConvLSTM) to extract the temporal features	* CNN-LSTM model was found to be the most suitable approach for investigation long-term activity recognition.
[25]	2020		sensor-based	They improved many models such as 1D CNN, a multichannel CNN, a CNN-LSTM, and multichannel CNN-LSTM	

**Table 2:** Activities of wireless sensor data mining (WISDM) [1].

Activity	Walk	Jog	Up	Down	Sit	Std
Samples	424,400	342,177	122,869	100,427	59,939	48,397
Percentage (%)	38.6	31.2	11.2	9.1	5.5	4.4

**Table 3:** Activities of UCI-HAR [1].

Activity	Walk	Up	Down	Sit	Std	Lay
Samples	122,091	116,707	107,961	126,677	138,105	136,865
Percentage(%)	16.3	15.6	14.4	16.9	18.5	18.3

**Table 4:** Classification of the confusion matrix on the WISDM.

Activities	Down	Jog	Sit	Std	Up	Walk	Precision	F1
Down	739	2	0	0	34	5	0.94	0.96
Jog	0	2576	0	0	8	0	**0.99**	**0.99**
Sit	0	0	395	20	1	2	0.94	0.97
Std	0	0	2	353	1	0	0.99	0.97
Up	20	4	0	0	855	0	0.97	0.95
Walk	6	2	0	0	14	3197	**0.99**	**0.99**
Recall	0.96	**0.99**	**0.99**	0.94	0.93	**0.99**		
Accuracy	98.53%							
Kappa	0.98							

The result marked in bold refers to the results that achieve the best classification of activities using different metrics.

**Table 5:** Classification of the confusion matrix on the WISDM using the proposed model, CNN-BiLSTM, as a feature extractor and support vector machine (SVM) as a classifier.

Activities	Down	Jog	Sit	Std	Up	Walk	Precision	F1
Down	646	0	2	0	30	2	0.95	0.96
Jog	1	2574	0	0	7	2	**0.99**	**0.99**
Sit	1	0	395	20	1	1	0.94	0.97
Std	2	0	0	354	0	0	**0.99**	0.97
Up	22	3	0	0	854	0	0.97	0.96
Walk	10	1	1	0	13	3194	**0.99**	**0.99**
Recall	0.94	**0.99**	**0.99**	0.94	0.94	**0.99**		
Accuracy	98.53%							
Kappa	0.98							

The result marked in bold refers to the results that achieve the best classification of activities using different metrics.

**Table 6:** Classification of the confusion matrix on the UCI-HAR.

Activities	Walk	Up	Down	Sit	Std	Lay	Precision	F1
Walk	494	2	0	0	0	0	0.99	0.99
Up	0	470	0	1	0	0	0.99	0.99
Down	2	10	407	0	1	0	0.96	0.99
Sit	0	3	0	449	36	3	0.94	0.91
Std	0	0	0	29	503	0	0.93	0.94
Lay	0	0	0	0	0	537	0.99	**1.00**
Recall	0.99	0.96	**1.00**	0.93	0.93	0.99		
Accuracy	97.04%							
Kappa	0.96							

The result marked in bold refers to the results that achieve the best classification of activities using different metrics.

**Table 7:** Classification of the confusion matrix on the UCI-HAR using the proposed model, CNN-BiLSTM, as a feature extractor and SVM as a classifier.

Activities	Walk	Up	Down	Sit	Std	Lay	Precision	F1
Walk	494	2	0	0	0	0	0.99	0.99
Up	0	465	5	1	0	0	0.98	0.99
Down	0	4	415	0	1	0	0.98	0.99
Sit	0	0	0	448	43	0	0.91	0.93
Std	0	0	0	24	508	0	0.95	0.94
Lay	0	0	0	0	0	537	**1.00**	**1.00**
Recall	**1.00**	0.98	0.98	0.94	0.92	**1.00**		
Accuracy	97.28%							
Kappa	0.96							

The result marked in bold refers to the results that are achieve the best classification of activities using different metrics.

**Table 8:** Comparison with other studies conducted on different human activity recognition (HAR) dataset.

Database	Ref.	Year	Used Technique	Accuracy (%)
UCF101	[13]	2015	GoogLeNet & VGG-16	91.40
	[19]	2015	TDD	91.50
	[22]	2016	Two CNN stream (VGG-16)	93.50
	[16]	2016	TSN	94.20
	[17]	2016	CNN	89.10
	[14]	2017	Two-stream-3D-ConvNet	93.40
	[23]	2018	CNN-LSTM	84.10
HMDB51	[19]	2015	TDD	65.90
	[16]	2016	Two CNN stream—iDT	69.20
	[17]	2016	TSN	69.40
	[14]	2016	CNN	65.20
	[23]	2017	Two-3D-ConvNet	88.40
ASLAN	[22]	2015	CONV3D-SVM	78.30
Sports 1M	[22]	2015	CONV3D-SVM	85.20
UCF-ARG	[10]	2020	Pre-trained CNN	87.60
Sound dataset	[21]	2020	CNN	87.20
HHAR	[3]	2020	Fusion ResNet	96.63
MHEALTH	[3]	2020	Fusion ResNet	98.50
WISDM	[1]	2020	CNN-LSTM	95.75
	[2]	2020	CNN	97.51
	Proposed	2020	CNN-BiLSTM	**98.53**
UCI	[15]	2016	CNN	93.75
	[20]	2018	CNN	95.31
	[20]	2018	CNN with stat. features	97.63
	[1]	2020	CNN-LSTM	95.80
	[2]	2020	Lightweight CNN	96.27
	Proposed	2020	CNN-BiLSTM	**97.05**

The result marked in bold refers to the results that are achieved by the proposed approach.

**Table 9:** Ablation study.

Experiment	Accuracy (%)	Precision (%)	Recall (%)	F1 Score (%)	Cohens Kappa
Six Conv. Layers at each level, work in parallel with two BiLSTM layers, then their output are concatenated	95.55	95.52	95.55	95.52	0.9465
Two Conv. Layers at each level, work in parallel with three BiLSTM layers, then their output are concatenated	75.97	78.93	75.97	75.27	0.7107
Two Conv. Layers, followed by BatchNormalization layer, work in parallel with two BiLSTM layers, then their output are concatenated	87.13	89.98	87.13	87.23	0.8456
One Conv. Layers, work in parallel with three BiLSTM layers, then their output are concatenated	88.63	88.99	88.63	88.51	0.8633

## Data Availability

The experiments have been carried out using sensor-based HAR datasets such as WISDM [31] and UCI [32] which are open for use in the research work.

## Abbreviations

The following abbreviations are used in this manuscript:

ADL	Activities of Daily Living
BiLSTM	Bi-directional Long Short-Term Memory
CNN	Convolution Neural Networks
DBNs	Deep Belief Networks
HAR	Human Activity Recognition
iDTs	improved Dense Trajectories
LSTM	Long Short-Term Memory
RBM	Restricted Boltzmann Machine
RNN	Recurrent Neural Network
SVM	Support Vector Machine
TDD	Trajectory-Pooled Deep-Convolutional Descriptor
TSN	Temporal Segment Networks
UCI	University of California, Irvine
WISDM	Wireless Sensor Data Mining
